# Chocolate Consumers and Lymphocyte-to-Monocyte Ratio: A Working Hypothesis from a Preliminary Report of a Pilot Study in Celiac Subjects

**DOI:** 10.3390/antiox8100440

**Published:** 2019-10-01

**Authors:** Anna Raguzzini, Giovanna Poce, Sara Consalvi, Elisabetta Toti, Francesca Palmacci, Mariangela Biava, Ilaria Peluso

**Affiliations:** 1 Research Centre for Food and Nutrition, Council for Agricultural Research and Economics (CREA-AN), 00178 Rome, Italy; 2 Department of Chemistry and Technologies of Drug, Sapienza University of Rome, 00185 Rome, Italy

**Keywords:** inflammation, immunity, diet

## Abstract

Background and aim: The aim of this work was to evaluate the relationship between platelet-to-lymphocyte ratio (PLR) and lymphocyte-to-monocyte ratio (LMR) with habitual consumption of dark chocolate in a group of celiac subjects in which chocolate consumption and lower neutrophil-to-lymphocyte ratio (NLR) association had already been observed. Additionally, due to the known anti-nutrient effect on iron absorption, we evaluated red blood cell count (RBC), mean corpuscular volume (MCV) and hemoglobin (Hb) values. Methods: Chocolate consumers and non-consumers were matched for sex, menopausal status, NLR values over the previously suggested cut off (2.32) for celiac patients, and co-morbidities. Results: Chocolate consumers had high LMR compared to non-consumers, whereas no differences were observed between chocolate consumers and non-consumers in RBC, MCV, Hb and PLR. However, similar number of subjects had PLR higher than the previously suggested cut off (143.7) for celiac disease. Conclusions: This preliminary report suggests a working hypothesis for larger studies aimed at establishing cut off values for LMR in celiac patients and the modulation of this marker by dietary antioxidants.

## 1. Introduction 

Chocolate contains both flavanols (epicatechin) and theobromine, non-nutrient bioactive compounds that can have potential pharmacological effects. Although it has been reported that a decrease in platelet activation in chocolate consumers can be ascribed to flavanols [1,2], Rull et al. [3] suggested that after six weeks of consumption such effect is modulated by a flavanol-independent mechanism that is likely due to theobromine. Although some recent studies suggested that catechins [4] and cocoa butter [5] may influence metabolic features, the improvement in cardio-metabolic risk factors (glucose levels, blood pressure, lipid profile) induced by cocoa or pure epicatechin is still under debate [6,7,8,9]. Moreover, it has been demonstrated that regular consumption of cocoa can exert anti-inflammatory effects by lowering the activation of monocytes and neutrophils [10]. We recently observed that chocolate consumption is associated with a lower neutrophil-to-lymphocyte ratio (NLR) in celiac subjects [11], in particular in those with NLR values above the cut off suggested by Sarikaya et al. (2.32) [12], supporting the previously reported hypothesis that the efficacy of cocoa products depends on the basal inflammatory status [10].

Platelet-to-lymphocyte ratio (PLR) and lymphocyte-to-monocyte ratio (LMR) have also been proposed as markers of inflammation in celiac disease [13,14] and acute ischemic stroke [15,16,17], and peripheral arterial [18], cardiovascular [19,20] and autoimmune [21] diseases. In particular, decreased LMR has been associated with increased disease activity in rheumatoid arthritis [21]. 

Meta-analysis data reported an increase in some cardiovascular risk factors (total cholesterol and fasting glycaemia) after a long-term gluten-free diet (GFD) [22] and a significantly heightened risk of venous thromboembolism among patients with celiac disease (CD) [23]. The aim of this work was to evaluate the relationship between both PLR and LMR and habitual consumption of dark chocolate in previously analyzed celiac subjects [11]. Moreover, given its well-known anti-nutrient effect on iron absorption [24], we evaluated potential differences on red blood cell count (RBC), mean corpuscular volume (MCV) and hemoglobin (Hb) values.

## 2. Materials and Methods 

### 2.1. Selection of Subjects

We extracted the data from a recent study [11], approved by the Ethics Committee of the Comitato Etico Lazio 2 (n° 43.18: 4, 2018). Written informed consent was obtained from all the participants in accordance with the Italian law (number 76/2008) and all procedures involving human subjects complied with the Declaration of Helsinki as revised in 2000. 

Chocolate consumers (1–3 times/week, *n* = 13) included celiac subjects all with Marsh III at diagnosis (2 men and 11 women). The chocolate non-consumers were selected within celiac subjects with Marsh III at diagnosis, matching for sex, menopausal status (3 in each group), NLR values over the cut off suggested by Sarikaya et al. [12] (4 in each group) and co-morbidities (2 thyroiditis, 1 allergy, 1 autoimmune disease: vitiligo/psoriasis). The two groups had similar smoking (3/10) habits.

The physical activity level (PAL), was evaluated by the International Physical Activity Questionnaire (IPAQ) and the adherence to Mediterranean diet was assessed with two different scores (the Mediterranean Diet Score: MDS 14: 14 items, each 0–1 score; and the MEDScore: Score 55: 11 items, each score range 0–5) as previously described [11]. In addition, sub-scores were calculated as follows: coherent (CO, high consumption of olive oil, fruits, vegetables, legumes, and fish and low consumption of red meat; ranges: 0–7 MDS CO7 and 0–30 Score CO30), incoherent (IC: wine and white meat, high consumption for MDS and low consumption for Score; ranges: 0–2 MDS IC2 and 0–10 Score IC10) and different (D: high consumption of nuts and Mediterranean sauce; and low consumption of butter, carbonated beverage and sweets for MDS; high consumption of unrefined cereals and potatoes, and low consumption of full dairy products for Score; ranges: 0–5 MDS D5 and 0–15 Score D15) [11].

### 2.2. Statitical Analysis 

Data were expressed as means with standard error mean (SEM) (Normality Test (Shapiro–Wilk) passed, two tailed T Test applied) or median (25%, 75%) (Normality Test (Shapiro–Wilk) failed, Rank Sum Test applied). The correlations (Spearman correlation) were analyzed between the parameters of interest.

## 3. Results 

Selected groups were similar in age, years at gluten-free diet (GFD), body mass index (BMI), systolic (SBP) and diastolic (DBP) blood pressure (mmHg) (Table 1). Overall (*n* = 26), platelets’ count (P) were inversely related to PAL (metabolic equivalent of task: MET) (coefficient of correlation –0.397, *p* = 0.044), which correlated both with Hb (coefficient of correlation 0.548, *p* = 0.004) and MCV (coefficient of correlation 0. 509, *p* = 0.008). On the other hand, no differences were observed in PAL (Table 1), P and markers of anemia (RBC, MCV and Hb) between chocolate consumers and non-consumers (Table 2).

**Figure 1:**
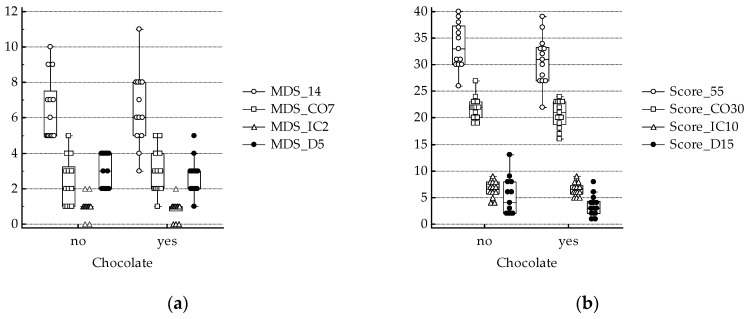
Adherence to Mediterranean diet (**a**) MDS 14 and sub-scores: coherent (CO7), incoherent (IC2) and different (D5); description of what is contained in the first panel; (**b**) Score 55 and sub-scores: coherent (CO30), incoherent (IC10) and different (D15). No significant differences between chocolate consumers and non-consumers.

We recently reported that NLR correlated with Score 55 and Score CO30 [11]. In the present study, although N correlated with Score 55 (coefficient of correlation 0.393, *p* = 0.046) and Score D15 (coefficient of correlation 0.424, *p* = 0.031) and the latter was also related to white blood cell count (WBC, coefficient of correlation 0.536, *p* = 0.005), significant differences between groups were not found for WBC and leucocytes’ subset counts (N: neutrophils, M: monocytes, L: lymphocytes; Table 2), as well as for adherence to Mediterranean diet (Figure 1). 

**Figure 2:**
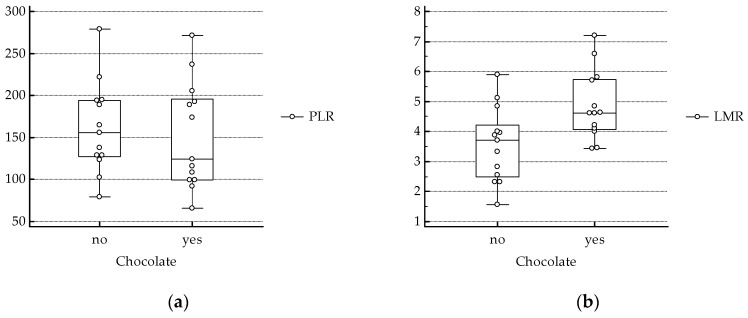
(**a**) PLR: platelet-to-lymphocyte ratio. No significant differences between chocolate consumers and non-consumers. (**b**) LMR: lymphocyte-to-monocyte ratio, Normality Test (Shapiro–Wilk) passed, two tailed T Test (chocolate yes versus no: *p* = 0.01).

On the other hand, NLR, lower in chocolate consumers [11], correlated with PLR (coefficient of correlation 0.588, *p* = 0.002), and was inversely related to LMR (coefficient of correlation −0.696, *p* < 0.001) (Figure 2).

## 4. Discussion and Conclusions

In this study we observed higher values of LMR in celiac subjects who consumed chocolate compared to non-consumers, whereas no significant differences were found in markers of anemia and PLR. One of the criteria for inclusion was the same number of subjects with NLR values over the cut off suggested by Sarikaya et al. [12] in the two groups. Consistent with the high correlation between PLR and NLR, similar number of subjects (6/13 consumers, 7/13 non-consumers) had PLR higher than the suggested cut off (143.7) for celiac disease [13,14]. Therefore, we did not observe significant differences in PLR between the two groups. Interestingly, while lower LMR and higher NLR were associated to short-term (30 days) mortality in patients with acute pulmonary embolism, PLR was not significantly different between survivors and non-survivors [25]. This suggests that PLR is a less sensitive marker of inflammation compared to NLR and LMR.

A lower LMR value is also an independent predictor of poor prognosis of some diseases and their outcomes. The suggested cut off values were 3.48 for neurologic impairment after acute ischemic stroke (AIS) [15], 3.38 for well-developed coronary collateral circulation in patients with stable coronary artery disease [19], 3.1 for critical limb ischemia in peripheral arterial occlusive disease [18], 2.99 for stroke severity and prognosis [16] and 2.0 for the six-month all-cause mortality in patients with acute heart failure [20]. Moreover, LMR has been suggested as a useful marker for stroke-induced immunosuppression [17]. Among AIS patients (on day one and seven), it was significantly lower in those also affected by pneumonia or urinary tract infection [17]. A meta-analysis of studies on celiac disease also suggested that it is associated with an increased risk of pneumococcal infection [26].

Furthermore, in rheumatoid arthritis, LMR was inversely correlated with inflammatory and immune-related markers (including PLR, NLR, immunoglobulin (Ig)A and IgM) and positively correlated with nutritional status markers (such as Hb and albumin) [21], suggesting a relationship between diet and autoimmune diseases. In this context, in the animal model of adjuvant arthritis in rats, a cocoa-enriched diet decreased antibody concentration and the T-helper (Th) lymphocytes and prevented the decrease in the proportion of regulatory T-cells in blood [27].

The work presented here is a preliminary study and has some limits. First of all, although sweets consumption (included in the MDS 5) did not differ between groups and the standard portion size was 30 g, we could not collect any information about either the percentage of cocoa in the consumed dark chocolate or food preference. The latter is the main factor driving food intake and choice and depends on many factors, including individual differences in human perception of sweetness partly due to polymorphisms in the taste-related genes TAS1R2/TAS1R3 [28,29,30,31] and interactions between bitter and sweet taste systems [32]. It is well known that in cacao polyphenolic compounds show both bitter and astringent characteristics, with a rejection threshold depending on percentage of cocoa in chocolate [33]. However, products with a psychoactive effect (such as coffee and chocolate) are generally preferred, regardless of their bitterness, and traits of consumers and health concerns could affect food preferences of bitter in vegetable foods [34]. Moreover, the relationship between TAS1R2/TAS1R3 and intake of sweet-tasting foods may be different across different populations [35,36]. In this context, chocolate accounted for 25%, 18% and 8% of flavanols, flavonoids and total polyphenols intake in Poland [37], but only for 7% of flavanols intake in Italy, where the main dietary sources of total polyphenols are nuts, followed by tea and coffee [38]. Moreover, other typical foods of Mediterranean diet, such as fruits and vegetables (including beans), contain high levels of polyphenols [38]. However, in the present study no differences were observed in sub-scores of Mediterranean diet adherence, including fruits, vegetables, legumes and nuts.

In conclusion, despite this pilot study’s low number of subjects and their low to moderate chocolate consumption (1–3 times/week), this preliminary report suggests a working hypothesis for larger studies aimed at establishing cut off values for LMR in celiac patients and its modulation by cocoa antioxidants.

## Figures and Tables

**Table 1:** Characteristics of subjects.

Chocolate	YES (*n* = 13)	NO (*n* = 13)
Men/women	2/11	2/11
Age (years)	44.0 ± 3.7	36.8 ± 3.5
GFD (years)	2 (1–10)	2 (1–7)
PAL (MET-min-week)	6166 ± 891	7518 ± 1379
BMI (Kg/m^2^)	22.1 ± 0.5	22.2 ± 0.6
SBP (mmHg)	116.9 ± 3.1	108.4 ± 3.1
DBP (mmHg)	72.5 ± 2.4	69.8 ± 4.0

GFD: gluten-free diet; PAL: physical activity level; MET: metabolic equivalent of task; BMI: body mass index; SBP: systolic blood pressure; DBP: diastolic blood pressure. Data are expressed as mean and SEM (Normality Test (Shapiro–Wilk) passed) or median and interquartile range (25%–75%) (Normality Test failed).

**Table 2:** Hematological parameters.

Chocolate	YES (*n* = 13)	NO (*n* = 13)
WBC (10^3^/microL)	6.2 (4.5–6.8)	5.4 (4.5–9.0)
N (10^3^/microL)	3.1 (2.2–4.3)	3.5 (2.2–5.8)
M (10^3^/microL)	0.36 (0.32–0.54)	0.44 (0.37–0.66)
L (10^3^/microL)	1.9 (1.4–2.4)	1.7 (1.1–2.0)
P (10^3^/microL)	275.4 ± 14.8	253.8 ± 19.0
RBC (10^6^/microL)	4.7 ± 0.1	4.7 ± 0.1
MCV (fL)	85.5 (77.5–87.3)	88.4 (82.9–92.3)
Hb (g/dL)	12.5 ± 0.6	13.5 ± 0.3

WBC: white blood cell count; N: neutrophil count; M: monocyte count; L: lymphocyte count; RBC: red blood cell count; MCV: mean corpuscular volume; Hb: hemoglobin. Data are expressed as mean and SEM (Normality Test (Shapiro–Wilk) passed) or median and interquartile range (25%–75%) (Normality Test failed).
